# Patient Experiences and Expectations Regarding Erectile Dysfunction Education Prior to Rectal Cancer Surgery: A Qualitative Study

**DOI:** 10.1007/s13187-025-02647-6

**Published:** 2025-05-15

**Authors:** Sebastian Borgund Hansen, Siv Fonnes, Birthe Thing Oggesen, Susanne Vahr Lauridsen, Jacob Rosenberg

**Affiliations:** 1 Department of Surgery, Center for Perioperative Optimization, Copenhagen University Hospital – Herlev and Gentofte, Borgmester Ib Juuls Road 1, 2730 Herlev, Denmark; 2 Copenhagen Sequelae Center CARE, Capital Region of Denmark, Department of Surgery, Herlev and Gentofte Hospital, University of Copenhagen, Borgmester Ib Juuls Road 1, 2730 Herlev, Denmark; 3 WHO-CC/Clinical Health Promotion Centre, the Parker Institute, Bispebjerg and Frederiksberg Hospital, University of Copenhagen, Norde Fasanvej 57 Road 8, Entrance 19, 2000 Frederiksberg, Denmark; 4 Department of Clinical Medicine, University of Copenhagen, Blegdamsvej 3B, 2200 Copenhagen N, Denmark

**Keywords:** Preoperative care; Qualitative research; Erectile dysfunction; Rectal neoplasms

## Abstract

Erectile dysfunction is common after surgery for rectal cancer with a prevalence of moderate to severe erectile dysfunction up to 35%. Preservation of sexual function is regarded as a core outcome in colorectal surgery. We wanted to investigate male patients’ perspectives on preoperative education about the risk of erectile dysfunction after surgery for rectal cancer. Using an interview guide, we performed individual semi-structured interviews with male patients who had surgery for rectal cancer within the past 3–12 months. The interviews were transcribed, condensed, coded, and analyzed with inductive qualitative content analysis. We interviewed 13 male patients. Their perspectives were condensed in four main themes: importance of a partner to be present at the preoperative counseling; information as crucial; wishful thinking as a coping strategy; and the need for direct information to avoid unrealistic expectations. Patients described general gratitude across the entire process but a lack of follow-up regarding erectile function. Surgeons should actively engage in a discussion with patients regarding the risk of developing erectile dysfunction. Importance might differ since patients have different preoperative erectile functions and different postoperative needs. A partner or close relative should participate in the preoperative counseling.

## Introduction

Rectal cancer affects around 700,000 people globally each year [1]. Advancements in surgical, oncological, and perioperative care have improved cancer survival rates. This has resulted in a notable shift in focus towards quality of life after surgery and not merely surviving the cancer diagnosis [2], entailing more emphasis on patient-reported outcome measures (PROMs) to quantify the quality of life issues faced by survivors [3]. One of the late complications in rectal surgery that has gained increased recognition is erectile dysfunction, which is also regarded as a core outcome of rectal surgery [4]. It is hypothesized that surgery, as well as chemo- and radiotherapy, independently poses a threat to the normal physiological function of the penis [5, 6]. Erectile dysfunction can have profound psychological, emotional, and relational implications [7]. While a few studies have explored patients’ experience of erectile dysfunction post-surgery, it remains unclear whether postoperative erectile dysfunction is adequately discussed before surgery and if it concerns patients while facing a cancer diagnosis [8, 9]. Additionally, it is important to recognize that the preservation of erectile function might not impact all patients similarly, and some patients will have erectile dysfunction preoperatively—underlining the inherently subjective nature of sexuality [10].

We qualitatively sought to explore male patients’ perspectives and thoughts regarding preoperative education about erectile dysfunction when interviewed within the first year after surgery for rectal cancer.

## Methods

This qualitative interview study was reported according to the COnsolidated criteria for REporting Qualitative Research (COREQ) guideline [11].

We included male patients who had surgery for rectal cancer within the past 3–12 months, who were able to talk and understand Danish, and who gave informed consent. We excluded patients with no preoperative erectile function. We only interviewed patients with some erectile function before surgery as this group of patients might have a fear of losing their function after surgery. Both patients with and without postoperative erectile dysfunction were included. We recruited patients using the principle of purposeful sampling with maximum variation [12]. The sample size was determined based on theme saturation [13].

Eligible patients from Copenhagen Sequela Center CARE (Colon-, Anal, and REctal cancer) at Herlev Hospital, Denmark, were contacted by telephone. They were informed about the aim of the study and their rights regarding participation. If they accepted, a date was set for the interview. No other formal relationship was established before the study commencement. They were informed that no recordings or transcripts would be passed on to third parties, that data and quotes were anonymized, and that data would be deleted after publication of the study. Oral informed consent was given before inclusion. According to Danish legislation, interview studies are exempt from formal ethics approval [14]. The Danish Data Protection Agency approved the study (P-2024–15586). Patients were informed that the interviewer was a medical student with a scientific interest in patients with rectal cancer, their treatment, and their life after surgery, and especially concerning erectile dysfunction. They were also informed that the research was conducted to improve patient information in the outpatient clinic and for future patients.

All interviews were conducted by the first author. At the time of the study, the interviewer was employed as a researcher at the Copenhagen Sequelae Center CARE. The researcher had little training in interviewing but was supported by the author group having extensive experience with qualitative studies. The telephone interviews were carried out between January through September 2024. In advance, all patients were urged to sit in a place where they would feel comfortable talking about this subject, preferably alone.

A semi-structured interview guide with open-ended questions was developed in collaboration with the co-authors. The interviewer asked broad questions to illuminate the research question. The interview guide was pilot-tested, adapted, and corrected throughout the series of interviews in an iterative process [15]. No repeat interviews were carried out. The research employed audio recordings to collect the data; no visual recordings were performed. The interviews were conducted and afterwards transcribed verbatim in their full length. Data saturation was defined as saturation of themes [13]. To define saturation of themes, we employed a strategy in which 10 initial interviews were conducted. A stopping criterion was established, with further interviews continuing until no new themes emerged after three additional interviews [16]. Transcripts were not returned to the patients for comments.

A qualitative interview design was adopted to obtain in-depth knowledge about patients’ thoughts, experiences, and attitudes toward preoperative information on erectile dysfunction. An inductive approach was applied. The qualitative methodological framework was that of content analysis [15]. Two of the authors were involved in the coding of the data (SBH and SVL). The coding process followed the steps of content analysis and employed a strategy involving condensing sentences into shorter quotes encapsulating their central meaning, each tagged with a corresponding code [15] as depicted in the coding tree in Table 1. These codes were then organized into categories and further broken down into subcategories in an iterative process, see Table 2. Through this process, connecting themes emerged, capturing meanings across categories.

**Table 1:** An example of how quotes were condensed into first meaning units, then condensed meaning units, and lastly codes

Meaning unit of short quotes	Condensed meaning unit	Code
“Imagine that roller coaster, that you’re in there, now I’m going to die, now you’re going to get a stoma and all sorts of things, and then now at least you’ll survive. I wouldn’t wish that roller coaster on anyone. In any way.”	Feeling of emotional imbalance and expectations about the future	Emotional turmoil
“Well, if nothing else had changed except for me being informed earlier, then no. Basically, the choice I was given was either you get the full package surgery, or you die. I mean, it’s still an easy choice. But you had to choose one.”	Perception of treatment options and decision-making	Treatment, decision-making, and outcomes

**Table 2:** Examples of how codes, categories, and themes are formed from the content analysis

Codes	Category	Theme
Communication and support options	Treatment, decision making, and outcomes	Information about erectile dysfunction as crucial
Role of companionship		
Supportive companion		
Shared decision-making in healthcare	Sexual health and function	
Lack of discussion about sexual health		
Male libido		
Emotional turmoil	Patient experience and emotions	
Uncertainty		
Emotional impact of cancer diagnosis and anticipation of surgery		
Presentation of treatment outcomes	Communication and information	
Communication style		
Surgeon’s focus		

## Results

We approached 14 patients regarding the interview study. No patients declined participation or withdrew consent; however, one patient could not be included due to language problems. The median age of the patients was 65 years (range 48 − 78), and the median time since surgery for rectal cancer was 5 months (range 3 − 12). Most patients (77%) had a partner at the time of the interview. A total of 46% of the patients had diabetes or hypertension (three had diabetes, three had hypertension). The interviews took a median of 26 min (range 16 − 54). After coding of the initial 10 interviews, data saturation was achieved. We interviewed three more patients without any novel themes arising, totaling a number of 13 patients interviewed.

The patients’ reflections on the preoperative information regarding erectile dysfunction after surgery for rectal cancer can be encapsulated in four themes: importance of a partner to be present at the preoperative counselling; information about erectile dysfunction as crucial; wishful thinking as a coping strategy; and the need for direct information to avoid unrealistic expectations.

### Importance of a Partner to Be Present at the Preoperative Counselling

All but one patient brought a partner to the preoperative counselling. They unanimously recommended bringing a partner for several reasons. First and foremost, it is easier to remember what had been said in the conversation.

I had my wife with me. It’s definitely an advantage. I mean, it’s clearly an advantage. Four ears hear more than two, right? You’re emotionally challenged in such a situation. That’s how it’s been with all the significant conversations I’ve had there. It’s really a good thing, isn’t it? Because she remembered things that had slipped past me, and vice versa (ID 5, 63 years old).

Additionally, the patient and the partner would be able to discuss what had been said. The nature of the conversation might be tense. Having a partner present allows the patient to share emotions that might arise after such counseling.

Yeah, so we also talked about the precise wording afterwards. And I wouldn’t remember that if I didn’t have someone with me. Then I might have missed some of it. Because it’s a tense situation, right? You can just go through it afterwards, process it. It’s a big advantage. (ID 4, 76 years old).

Patients also reflected that a shared sorrow is less heavy.

But yes, having a partner is good. Also, because you hear something, but you’re also in a kind of vacuum or a bit of shock or something. It all depends on where you are in the process. I would always recommend having someone with you (ID 7, 52 years old).

By informing not only the patient but also the partner, it enables the patient to talk more readily about postoperative difficulties such as erectile function as illustrated in the following quote.

It’s good to have your partner with you because it’s something that affects both of us. So I think my partner should be involved in saying… or helping with the decision about what we do. (ID 12, 61 years old).

### Information About Erectile Dysfunction as Crucial

Patients would choose surgery even if they were informed that erectile dysfunction was guaranteed. However, patients often described postoperative concerns regarding a decrease in erectile function, that they did not expect after the preoperative conversation.

It’s the operation I need to survive. Let me put it this way, it definitely did not stop me. I can guarantee you that. If they had told me that I would get a stoma, right up there where you can’t hide it. Your penis won’t work. And in that case… Well, you’ll also need a bag for urine. So your body won’t work at all. But we need to go through with an operation because there’s a risk you could die from it. I still think I would have said yes. (ID 3, 51 years old).

No, I don’t remember that, I actually don’t remember exactly when I talked about erectile issues… I can barely even remember talking about them – but one does have erectile problems. (ID 13, 71 years old).

Patients also emphasized, that while the surgeon could not predict whether or not a specific patient might experience erectile dysfunction postoperatively, it would be beneficial to mention the risk and availability of treatment options.

He mentioned that there could be problems afterwards… Exactly things like getting an erection and going to the toilet, peeing, and stuff like that. It was also mentioned that there was a possibility of late complications and then you could do different things. What those different things were, we didn’t talk about. It wasn’t really a focus at that time. (ID 6, 48 years old).

### Wishful Thinking as a Coping Strategy

Patients employed a coping strategy involving wishful thinking regarding surgery with a tendency to downplay the magnitude of surgery or which late complications they might experience. An example of a train of thought could look like this: The cancer is the main problem, surgery is the solution, and when surgery is completed, I will be healthy again. Hence, a rather uncritical attitude was displayed towards the surgery being the solution to the problem.

I fully trusted them. Actually, I haven’t been afraid of this. And that sounds a bit silly, and you’re allowed to scold me. But for me, this operation was very, very minor. (ID 7, 52 years old).

Some patients made statements dealing with alternative realities or magical objects, stating that only the surgeon could foresee which possible complications might arise. Other patients entertained the idea of alternative realities.

But of course, when you’re sort of playing with the idea of, what if… That’s why I mentioned that if one had a crystal ball, then one could have said, no, I don’t need that. And then one could have avoided all those symptoms, and such. (ID 2, 69 years old).

Finally, some patients displayed great trust in the surgeons, choosing to believe that their suffering throughout the process would ultimately result in a positive outcome.

No, but I would say that I have been very fearless. Open to the fact that it is indeed an operation one has to go through. It will take some time and it will hurt and all sorts of things. But it’s for my own good. (ID 8, 57 years old).

### The Need for Direct Information to Avoid Unrealistic Expectations

Patients described how factual and direct information leaves less room for doubt even though the information might seem tough. Giving concrete information and recommendations might empower patients, offering a sense of control over their health, considering the helplessness some have felt upon receiving their cancer diagnosis.

I could have really used some support. It’s been a little frustrating… I didn’t have much information about how it was going to go… I feel like I’ve been kind of on my own with it. (ID 11, 64 years old).

Well, I think we talked a bit about it, my girlfriend and I. She still found some of the information to be quite tough. About some of the things, right? But I mean, I think it was important to get the information and understand the seriousness, right? (ID 6, 48 years old).

Some patients who were disappointed about their sudden decrease in erectile function also underscored the unintentional harm that surgeons might impose on their patients by “sugarcoating” the information.

I have expressed myself, and I still stand by it, that I want straightforward information. Not all that packaging and stuff, just give it to me so I have something to relate to, so these things don’t catch me off guard. Give it to me straight. (ID 3, 51 years old).

## Discussion

In this qualitative study, we found that four themes were consistent in the discussion of preoperative information on erectile dysfunction following surgery for rectal cancer. Patients generally agreed that partners or close relatives should be present at the preoperative education. Patients prioritized surgery over function but wished for more information about the postoperative risk of erectile dysfunction. Patients’ reflections underscore the necessity for surgeons to give realistic expectations about the future and to open a door for conversations about possible treatment options after surgery. Patients wished for concrete information about possible erectile dysfunction after surgery but described general gratitude across the entire process.

We found that all patients recommended that a partner or close relative attend the preoperative counseling. Reasons included that four ears hear better than two, and that a shared sorrow is less heavy. One mixed-method study found that in preoperative counseling before radical prostatectomies, spouses played a complex role and that surgeons should interact with both partners to identify the patient’s true needs [17]. This could also be the case for patients undergoing rectal cancer surgery and underscores the complex nature of the counseling. Additionally, involving a close relative in the preoperative counseling could ease the patient’s ability to communicate their needs and limitations postoperatively [18]. Thus, the support network not only aids in decision-making but also facilitates effective communication during the recovery process.

Though erectile dysfunction following surgery for rectal cancer is very common and has a prevalence of between 35 and 84% [19, 20], one prospective cohort study found that sexual activity, interest, and enjoyment remained relatively stable after surgery for rectal cancer [21]. This prioritization is readily explained. However, we found that most of the patients considered it crucial to be informed about this possible complication of the surgery. Since men of that age might have some pre-existing sexual impairment [22], a further reduced function might hurt more. It is also worth mentioning that while the patients indicated a general lack of information regarding postoperative erectile dysfunction, we cannot definitively determine whether they received this information. The patients were interviewed between 3 and 12 months after their surgery, and more than half of them did not recall being informed about the risk of developing erectile dysfunction during preoperative counselling. Some patients discussed this issue with the stoma nurse. It is unclear whether the patients were not informed or if they simply forgot or disregarded it. However, a Canadian qualitative interview study found that 47% of patients interviewed after surgery did not recall any discussion regarding risks to sexual function, such as impotence [23]. Surgeons might not discuss this issue, potentially considering it irrelevant due to the age of the patients and assuming they know what patients are interested in hearing [24]. It is definitely possible that the topic was not mentioned at all, that the surgeon did not emphasize it sufficiently, or that the patients simply forgot or overheard the information provided. This discrepancy highlights a gap between the manifest content of the interviews, where patients express a lack of information, and the latent content, which suggests an underlying desire for better communication and understanding to help manage their postoperative expectations and reduce the need for unrealistic coping strategies.

Upon receiving a cancer diagnosis, patients experience considerable psychological distress. Various uncertainties exist regarding the future, and colorectal cancer surgery encompasses many of these. Patients seek answers to questions regarding life expectancy, whether they will have to keep their stoma, which late complications might arise, and questions concerning whether they will be free from cancer or if any metastasis is present [25]. In the discussion with the surgeon, patients are eagerly seeking answers that can attempt to ease this existential uncertainty. In the context of this study, it is the surgeon’s responsibility to actively engage in a discussion with the patient about risk of erectile dysfunction after surgery as well as possible treatment regimens. This discussion can be personalized to fit individual patient needs, since it will not be important for all patients. Concerning the patients’ wishful thinking strategy to cope with the surgery and postoperative course, a review found that physicians tend to let the treatment plan dominate early discussions on cancer treatment, thereby deflecting attention from discussions of prognosis [24]. Additionally, the authors noted that surgeons tended to downplay potential complications, offering minimal detail regarding their severity or long-term consequences [24]. This might explain why patients have an overly optimistic idea of the peri- and postoperative course leading to a wishful thinking as a form of coping strategy. The wishful thinking observed among patients in this study may serve as a means to maintain hope during a life-changing event. However, it stresses the importance of continued education and realistic expectation-setting during preoperative counselling sessions. By providing patients with accurate information about the prevalence of erectile dysfunction and preparing them for potential challenges post-surgery, healthcare providers empower patients to seek appropriate support postoperatively [26].

The strengths of this qualitative study are firstly that we followed the reporting guideline COREQ [11], ensuring transparency in the reporting. Secondly, we included both patients with and without postoperative erectile dysfunction but all with some function before surgery. This inclusive sampling ensures a greater level of external validity. Thirdly, we conducted content analysis—a common method within qualitative methods [27]. Finally, the use of phone interviews offered an advantage by creating a less intimidating environment for patients, encouraging more candid responses about this intimate and personal subject [28]. However, there were also some limitations to this qualitative study. Limited experience in qualitative techniques from the interviewer might have constrained the quality of the interviews. To mitigate this, ongoing discussions with an expert in qualitative methods within the author group was employed, enabling the detection and correction of errors. Continuous support and guidance provided by the author group who listened to interviews and gave feedback also limited the risk of methodological error and ensured consistency and reliability in the data collection. The study did not include a follow-up procedure of gathering feedback from patients on themes. Since there was no visual input missing evaluation of eye contact and body language, this could be a limitation to the study—however, some patients find that the distance created by the phone interview provides a positive sense of protection when sharing [28]. In addition, the time between the first phone call and the interview allowed time to consider involvement, keeping in mind the taboo nature of the subject.

There is a need for better and long-lasting education of patients undergoing surgery with possible late complications such as rectal cancer surgery. This may be achieved by supplementing verbal information with other types of information that can be reassessed at a later time point. In a review including 145 articles, researchers found that the level of perceived knowledge among rectal cancer survivors with a stoma was increased with 95% of patients feeling more informed after reading a booklet [29]. Addressing sexuality and erectile dysfunction in such a booklet could serve as a foundation for patient education and discussion. This resource can be regularly updated with new evidence. An alternative to booklets or brochures is videos. Educational or explanatory videos about the procedure or about what to expect after the surgery can better prepare the patient to engage in discussions with the surgeon [30, 31]. However, it should be stressed that the information should be given with the possibility of feedback from the patient since not all patients have erectile function before surgery, and those who might already have a loss of function will be more vulnerable. Surgeons should therefore engage actively in this. This prerequisite education of the surgeon on the matter with regards to the prevalence of this complication and possible treatments.

In conclusion, male patients find it crucial to receive detailed preoperative education about erectile function in relation to surgery for rectal cancer. However, given that patients have diverse needs, a personalized approach is essential both in content and delivery. Surgeons are responsible for addressing erectile dysfunction and engaging in this discussion if it is important to the patient. Additionally, patients emphasized the importance of involving their partners whenever feasible.

## Data Availability

Data are not available due to national legislation.
